# What Do Dentists and Dental Students Think of Oral Cancer and Its Control and Prevention Strategies? A Qualitative Study in Jazan Dental School

**DOI:** 10.1007/s13187-019-01609-z

**Published:** 2019-09-10

**Authors:** Mohammed Jafer, Rik Crutzen, Ibtisam Moafa, Bart van den Borne

**Affiliations:** 1 Department of Preventive Dental Science, College of Dentistry, Jazan University, Jazan, Saudi Arabia; 2 Department of Health Promotion, CAPHRI, Maastricht University, Maastricht, The Netherlands

**Keywords:** Oral cancer, Oral cancer screening, Early detection, Dental education, Curriculum, Risk factors, Patient education, Perception

## Abstract

Jazan region of Saudi Arabia has a high prevalence of oral cancer: 35% of cases. However, little is known about the view of dentists and dental students on possible factors that contribute to this high rate with scarce efforts regarding prevention. The study investigated possible factors related to oral cancer as perceived by dentists and dental students, using a qualitative approach. Six focus groups conducted in Jazan Dental School (JDS) and participants included dental students, interns, faculty members, and key decision-makers. All participants acknowledged oral cancer a serious public health issue in Jazan affecting all ages. The majority thought that tobacco is a major risk factor in this region, which can easily be obtained by individuals of any age or gender. Further, formal dental education in the country is not focusing on the local related risk factors. Participants agreed that practicing oral cancer examination and its risk factors education is related to clinicians’ attitude and skills. Several participants declared that no organized effort established toward oral cancer and its risk factors in the region. Cultural acceptance of oral cancer risk factors was mentioned as one of the challenges for patients. The findings indicate that dental education and training at JDS are not focusing on necessary related oral health issues and their risk factors in the community. Also, it casts a new light on the fact that dentists are not educating their patients because they lack the knowledge and the skill of health education and patients’ communication methods.

**Electronic supplementary material:**

The online version of this article (10.1007/s13187-019-01609-z) contains supplementary material, which is available to authorized users.

## Background

Oral cancer is a major dental public health issue in several developing countries [1]. Saudi Arabia has a high prevalence of oral cancer and the majority of diagnosed oral cancer cases are in the Jazan region [2]. The high consumption of tobacco products, particularly smokeless tobacco, is found to be a major risk factor for oral cancer [3]. Shammah is a type of smokeless tobacco that is consumed in Saudi [4] and increases the risk of oral cancer [5]. Additionally, smoking tobacco is also strongly associated with oral cancer [6] and highly consumed in Saudi and not limited to a specific gender or age, for example 34% of male and 11% of female secondary school students are smoking cigarettes or shisha, hookah which are synonyms for water pipes tobacco smoking [7].

A key issue related to oral cancer is the delay in diagnosing oral cancer cases, which was identified as a risk factor for mortality of such cancers [8]. The delay of oral cancer diagnosis leads to delay in treatment delivery and decreases the survival rate of patients [9]. Multiple factors contribute to the delay of oral cancer diagnosis, such as health care providers’ knowledge, which was found to be strongly associated with performing full oral cancer examination [10]. Also, health care providers are not preforming full oral cancer examination but mainly focus on previous existing health conditions [11]. In addition to health care providers’ behavior, patients’ behavior could contribute to such delay in oral cancer diagnosis. Delay in seeking medical care due to wrong interpretation of symptoms leads to delaying the detection of oral cancer [12]. Such behavior could be attributed to the low level of awareness among patients [13].

Incidence of oral cancer can be reduced through prevention of its risk factors. The cost-effectiveness of prevention and early detection has been documented [14]. Health care providers could help in reducing the oral cancer burden by increasing their patients’ awareness of oral cancer risk factor. Furthermore, early detection of oral cancer through full oral cavity clinical examination is critical. The earlier the detection of oral cancer is, the better is the outcome of the treatment [15]. Therefore, dentists play an important role in detecting oral cancer and in educating individuals who are at risk of oral cancer.

The aim of the present qualitative study was to obtain insights regarding the factors that were perceived to contribute to the high prevalence of oral cancer in Jazan region of Saudi by dentists and dental students, who are the main future dentists in Jazan region. While the broader aim was to assess oral cancer practice in Jazan, which has a high rate of oral cancer, using a bottom-up approach. Therefore, the first step toward assessing oral cancer practice was to investigate such practice among the first line of oral health care providers.

## Methods

The present study investigated factors perceived to be related to oral cancer using a qualitative approach via the focus group discussion (FGD) method and was reported according to the consolidated criteria for reporting qualitative research (COREQ) [16]. All FGDs were facilitated by a male main facilitator (MF), who was the principal investigator, and a female co-facilitator (CF). Both are dental public health specialists and faculty members of the dental school at Jazan University, Saudi, and Ph.D. candidates at Maastricht University, the Netherlands. The MF was trained in designing and conducting qualitative research. The MF and the CF had additional training in facilitating FGDs on two mock FGDs with participants similar to the targeted sample under supervision of an expert professor of health promotion from Jazan Applied Sciences School. Moreover, a mediator, who is an expert professor of health promotion from the Jazan Dental School (JDS), observed all FGDs to ensure that all topics were thoroughly discussed. All participants were dentists or students at the dental school of Jazan University. Participants were informed of the purpose of the focus group discussion and were willing to share their ideas and experiences. All discussions were observed by an expert professor of health promotion from JDS, who was independents of the study. Furthermore, the present study considered a serious dental public health issue, oral cancer, in Jazan region: an uninvestigated topic about which limited knowledge is available. The present study used a grounded theory methodology to explore the ideas and thoughts of JDS members and clinicians regarding oral cancer (see Fig. 1 in the ESM).

### Participant Selection

Initial purposive sample using a general invitation delivered to faculty members, interns, and students of JDS by clinics administration was obtained. A total of 14 participants were assigned to two FGDs as follows: the first FGD involved two male and one female faculty members, one male and one female interns, and one male student. The second FGD had two male and two female faculty members, one male and two female interns, and one female student. Initial analysis of the first two FGDs indicated the importance of adding certain individuals: academic administrators and oral cancer–related course directors, who could enrich the discussion on the topic, because of their direct involvement. The influence of the formal dental education and training at JDS seemed to be the main focus of previous FGDs; a theoretical sample was acquired as several key decision-makers in JDS were invited by the MF to participate in the third and fourth FGDs. Also, interns and students were invited to participate in those FGDs.

The third FGD included the dean of JDS and two directors of courses who were involved in oral cancer education, oral medicine, and oral radiology. A total of four male and one female faculty members participated in this FGD in addition to one male and one female interns and one male student. The fourth FGD included the academic vice dean and the head of quality and academic development department in JDS. It also included two course directors of courses with major involvement in oral cancer education, oral pathology, and oral surgery. A total of six male and two female faculty members with one male intern, and one male and one female students have participated in this FGD.

While analyzing the queried data, students’ and interns’ interactions in the previous four FGDs seemed to be fairly limited. Therefore, two FGDs consisting of only students and interns were established. This provided them with more space to interact independent of dental supervisors or trainers and to express freely their thoughts, ideas, and experiences. Furthermore, to comply with JDS administration inquiry, separate male and female FGDs were conducted. Each FGD included four students and four interns from JDS.

### Data Collection

Started on the 25th of December 2016, a total of 6 FGDs with the number of participants varying from 6 to 11 took place in JDS’s main faculty meeting room. A period of 2 months was the time difference between the three sampling phases described above (FGDs 1–2, 3–4, and 5–6) with 1 week apart between all phases. Besides the female students’ and interns’ FGD which was facilitated by the CF, all FGDs were facilitated by the MF and the mediator. A semi-structured FGD guide including ten open-ended questions (Appendix 1) was developed by the authors. The developed FGD guide was sent to three oral cancer specialists for content evaluation who agreed on limiting the topic to oral cancer without investigating pharynx cancer, which has difference risk factors compared with oral cancer. The FGD guide was thoroughly discussed with three specialized professors from JDS including the mediator who acknowledged the previously mentioned point and added that pharynx cancer was not prevalent in Jazan region of Saudi where the present study was going to be conducted. Furthermore, the FGD guide was piloted with a group of five faculty members from JDS and used at the training sessions of the MF and CF. In the last two FGDs, a short FGD guide (Appendix 2) which was developed from the original one specific to issues that had less students’ and interns’ contribution was followed. All FGDs were audio recorded and then transcribed by the MF and CF and cross-reviewed by each other. Mediator notes of each FGD were taken in considerations in terms of conducting the next FGD or analysis. All FGDs lasted around 2h each except the fourth FGD which lasted for two and half hours. The saturation of the data was reached after the fifth FGD. However, the sixth FGD, male students and interns, was conducted due to previous planning and confirmation of attending from participants, and to see whether the male perspective differed from the females.

### Data Analysis

All FGDs were in Arabic and all discussion materials were manually analyzed by MF and CF as follows: Manuscript and memos of the discussion were reviewed after each FGD by MF and CF and discussed with the mediator. Initial codes were then abstracted from data. All similar codes were summarized to representing focus codes which led to theoretical codes (Table 1 in the ESM). A coding tree was created to illustrate the coding process and to enhance understanding of the data (Fig. 2 in the ESM). All codes were supported by participants’ quotations without any form of referring to participants. Subsequently, each developed focus code was reported in the “Results” section with its major related initial codes and participants’ quotations. All discussion materials were translated into English by the MF and reviewed by CF and evaluated by all authors. The reason for conducting the interviews in Arabic is that this study has an international team whose members do not all speak English. All the participants are Arabic native speakers and they have preferred the interviews to be in Arabic language as they could express their thoughts better. Data analysis was done on the Arabic transcript as analyzing English-translated transcript was not a recommended option because data would be susceptible to deterioration and loss of valuable information. Besides, there was no software that could operate the Arabic language which left us with no option except to use the manual qualitative analysis approach.

## Results

The FGDs revealed five major themes representing participants’ ideas and thoughts regarding perceptions and thoughts about oral cancer and its causes. The first theme was related to the severity and distribution of oral cancer in Jazan region and possible factors regarding delay in diagnosis and was referred to as a public health issue. The second theme was behavioral and cultural related risk factors related to consumption of tobacco products and other local consumed crops, e.g., Ghat. These two themes reflected the acknowledgment of oral cancer and its risk factors seriousness in Jazan region. The third theme was curriculum influence, which indicated the impact of the Jazan Dental School’s curriculum on oral cancer’s recent and future related medical practice in the region. The fourth theme was clinicians’ behavior toward oral cancer, which reflected the clinicians’ opinion about the factors that may contribute to their behavior toward oral cancer related medical practices. Lastly, the fifth theme was the challenges and barriers that participants faced through their clinical practice related to oral cancer in Jazan region of Saudi.

### Public Health Issue

All participants acknowledged that oral cancer is a serious public health issue in the Jazan region that is affecting all ages: “I believe that oral cancer is a real problem in Jazan,” “… not only for old people but for young too.” They also mentioned that oral cancer has a high prevalence rate in the region, “According to the data available from Saudi records, this region has high rate of oral cancer patients.” According to some participants, the prevalence of oral cancer might be related to gender and specific geographical location in the region, “Oral cancer in Jazan got specific geographical and sex distribution … and affects women more than men.” Several participants stressed that the seriousness of oral cancer in the Jazan region is due to the delayed diagnosis of such cases, “I think it’s serious because people are going undetected for years ... and when they are finally diagnosed, it’s so late and condition is hopeless.” Participant suggested multiple factors that might contribute to this delay in diagnosing oral cancer cases in the region; the first factor is the lack of specialized medical centers where oral cancer cases could be referred to for diagnosis and treatment: “… a center for consultation in which dentists can refer the suspicious cases should be present in Jazan.” The second factor is the discrete number of specialized health care providers who are trained to deal with oral cancer cases: “to be honest, I’ve never seen any specialist in oral medicine conducted diagnosis of difficult cases during my years of education.” The third factor a participant brought up was the awareness among dental patients of oral cancer and its risk factors in the Jazan population: “… on community level, I think the low awareness regarding risk factors is the main reason for high rates of oral cancer.”

### Behavior and Cultural Related Risk Factors

The majority of participants thought that tobacco is a major risk factor, which is available in the region and could easily be obtained by individuals of any age or gender in the Jazan region: “…and some of them are of young age,” “females use Shammah and have more access to it in Jazan than males.” Furthermore, several participants thought that utilizing tobacco products is culturally accepted: “…using Hookah between young females is quietly common…and society may accept using Hookah among women.” It was mentioned by several participants that individuals with low socioeconomic status might tend to use smokeless tobacco, which is highly associated with oral cancer: “It’s a real problem that needs to be addressed, and it has a socioeconomic relation that should be investigated more.” Also, they thought that education level, working status, and location of an individual may contribute to the use of the tobacco: “… using Shammah depends on education, and if ladies work or not … the majority of them are coming from the rural areas and usually they are illiterates…most of oral cancer cases used Shammah.” According to several participants, another substance that is frequently used in the region is Ghat, which is always combined with tobacco products: “… here, it’s a common belief that who is chewing Ghat is smoking too, at least Hookah or cigarettes. But I have another one related to Shammah; people who chew Ghat usually tend to use Shammah at the same time.” They also believe that the consumption of tobacco could be more severe when it is combining with Ghat use: “…especially if he/she is chewing Ghat, he/she may use Shammah up to 20 times per day.”

### Curriculum Influence

Almost all participants agreed that one of the most important factors affecting their dental care practices is their formal dental education and clinical training. Participants also agreed that the local oral health issues must be part of their education and clinical training. Therefore, participants emphasize on incorporating oral cancer subject into Jazan Dental School curriculum, as they considered oral cancer a major oral health issue, which is more prevalent in Jazan in comparison with other regions within the country. Some respondents pointed out that because Jazan Dental School is one of the most recently established dental schools in the country (i.e., established in 2008). Also, its curriculum should be benchmarked against national and international dental schools, which may not necessary have similar local health issues: “… with high confidence, I will say that in our current curriculum, we are not taking regional issues in consideration and this is happening for other Saudi dental schools as well; because we need national and international bench-marks. And we don’t have a national one that represents the region.” In addition to the curriculum construction issue, respondents agreed that data related to oral health issues in the region are limited, and since most of the faculty members in the dental school are not from Saudi, they may not be informed of the region’s needs “… we need to change the curriculum, have sound studies and bring more studies and evidence so it can be included in curriculum… lectures have to be updated according to the area needs.” The discussions also showed that there is limited involvement of the local oral health needs in the construction of the dental educational material and clinical training which leads to limited exposure of the dental students to such needs: “…exposing the students during their studies is very weak. No expose of cases or referring.” It was mentioned that the didactic education on oral cancer and its related risk factors must be supported by adequate clinical exposures. Participants believe that such exposures could be obtained better in the more specialized hospitals and centers where students could observe and participate in examining, diagnosing and treating oral cancer cases: “…we were not exposed to clinical training with oral cancer cases… when I went to the ministry of health, we almost see patients every week....” Therefore, participants thought that collaborations with such organizations should be established to facilitate a satisfactory training for the dental school students.

Prevention of oral diseases is an essential component of the Jazan Dental School mission, as was stressed by several participants. For that, students and clinicians in the Jazan Dental School clinics expected to have adequate knowledge and training on risk factors of the region’s diseases. However, the methods, skills, and practices of educating patients on issues related to oral cancer and its risk factors are not part of the Jazan Dental School’s curriculum, “… I’m sure that no student has been exposed or taught about how to help oral cancer patients.” Also, dental students should be trained to accurately communicate health advices with their patients: “…I was facing problems in how to make sure that a patient gets the message. How can I insure that he understands what I want to tell him… but I have no experience in communicating with patients.” In addition to patients’ education regarding oral cancer and its risk factors, respondents agreed that dental schools should carry the responsibility of raising the local population awareness of regional oral disease and risk factors. However, they mentioned it is not the case at Jazan Dental School, yet “… but till the last year we didn’t go out or did program for community.” Participants also indicated that the only oral health education program was initiated by dental students with limited involvement from Jazan Dental School “… people don’t know the threats of Shammah and there are no official campaigns or anything educating the community by the dental school besides some student efforts.”

### Clinicians’ Behavior Toward Oral Cancer

Discussion revealed two major agreements among all participants toward practicing oral cancer screening and patients’ education of its risk factors. First, practicing or not practicing oral cancer examination and its risk factors education in their clinic was perceived as a behavioral issue rather than being limited to lack of knowledge “…the problem is behavior based but not knowledge.” Second, the reason of not screening for oral cancer is because they are not thinking of the consequence of such behavior: “…they are not taking it seriously*.*”

Furthermore, several antecedents’ factors were referred to be influencing their behavior. Among those factors are three main enabling factors: no clinical examination protocol, and absence of referring and tracking system. Lacking such a protocol and system that guides clinicians through their clinical practice in the dental school may contribute to such behavior: “…there are no clinical guidelines or manuals to follow in college.” Therefore, participants stressed the need of creating a protocol that takes into consideration Jazan region’s oral health issues such as oral cancer, which will ensure optimum clinical practice. Participants stressed that this protocol should include possible referring and tracking methods and pathways: “… I think we should have our own protocol in our college where there is a system that tells us how to deal with such cases, where to refer to and how to track these cases.”

In addition to the enabling factors, the participants discussed predisposing factors that might influence their behavior toward oral cancer practice. First, most of the participants revealed that they usually depend on previous dental examination and focus only on the patients’ main complains: “… we are confident that our diagnostic clinic here does that for us.” Based on interns’ and students’ discussions, the majority of the participants agreed that they mainly focus on the required dental procedures to pass their courses “… because of the stress of requirements and other related stuff, only the teeth appear visible for us while the rest of the mouth unintentionally disappears from our sight.” Other reinforcing factors were mentioned that influence the students’ behavior toward oral cancer examination and patients’ education of its related risk factors. For instance, students might skip some clinical examination steps because of the limited time in their clinics “… in oral diagnoses, there is not enough time for students for the diagnosis.” Another factor was the lack of specialized supervision in the comprehensive clinic where students are individually required to fully rehabilitate completed dental cases. Because there is no such supervision, students tend to neglect examining the full mouth and focus only on tooth-related issues: “… in a comprehensive course, no one cares about the soft tissue … in a comprehensive team, there is no supervisor from the oral medicine.”

### Challenges and Barriers in Clinical Practice

Participants addressed different kinds of challenges that may impact their practice regarding oral cancer examination and patients’ education of its related risk factors. For example, Saudi participants agreed that performing oral cancer examination on the opposite gender is uncomfortable and affects their ability to conduct a satisfying clinical examination: “… I find it very difficult for me to do the oral cancer screening on a female patient....” On the other hand, among non-Saudi participants, gender did not have a noticeable effect on the way they performed their clinical examinations; however, language did, if Arabic was not their mother tongue.

Other challenges were related to the cultural acceptance of the main oral cancer risk factors. For instance, some patients use Shammah as remedy: “… there was one female patient who had an ulcer in her tongue, her neighbor advised her to put Shammah on the ulcer so it heals fast. She showed up with a white lesion because of that advice.” Another harmful behavior is smoking Shisha/Hooka/Moasil, which is popular among youths including young females: “… using Hookah among young females is quite common and they won’t feel any embarrassment if you ask them.” Although most of the participants indicated that they have knowledge about the spread of such risk factors, the majority of them felt unequipped to deal with such behavior-related problems: “… we don’t know the clear steps for changing his/her behavior.”

## Discussion

Dentists’ formal education and training influence their practice of oral cancer examination and oral cancer risk factor education. Knowledge contributes to the behavior of dentists through different mediators such as attitude, subjective norms, and perceived control. Similar to a previous study, the present study indicates that dentists’ knowledge of the topic is necessary for preforming oral cancer examination and oral cancer risk factor education [17]. Therefore, future dentists may lack the required competencies for effective oral cancer practice as a consequence of insufficient attention to local oral health issues within their education and training [18]. Likewise, dentists’ attitude and skills regarding oral cancer practice are crucial in their engaging in such behaviors [19]. Results of the present study are in line with previously published findings in relation to dentists’ limited performance of full clinical oral cancer examination and patients’ education of oral cancer related risk factors [20]. If the faculty members do not perform full clinical oral cancer examination and patients’ education about its related risk factors, such behavior can be cross-transmitted to their dental students [21]. The dental students could be influenced by the significant others’ beliefs and behavior toward performing oral cancer examination and education of patients about oral cancer risk factors, such as by their fellow dental students and professors. Moreover, the perceived school support for the practice, and the degree of dental students’ motivation to comply with their fellow students’ and supervisors’ beliefs and behavior may influence the dental students as well [21].

In addition, the present study findings revealed that the clinical courses’ requirements for dental students may influence students’ behavior toward oral cancer examination and patient education as this may consume more time and perhaps have an effect on the patients’ retention. Previous studies were limited to investigating this regarding academic performance and stress [22, 23]. Hence, the findings of the current study are novel and need further investigation because it may require an adjustment of the clinical courses’ requirements and support of dental students-patients’ communication. Furthermore, perceived behavioral control toward conducting oral cancer examination and patient education constitutes an important determinant for succeeding in performing oral cancer examination and patient education as well [21]. Lacking of the related necessary skills to perform oral cancer examination and patient education of its risk factors contribute to not practicing [21]. On the other hand, cultural aspects such as reluctance of dentists or their patients toward oral cancer examination performed by the opposite gender, and different cultural backgrounds or non-Arabic-speaking dentists, may contribute to not fully practicing oral cancer examination and patients’ education behavior by dentists [24]. In the present study, several participants mentioned that they encounter difficulty examining the opposite gender, particularly local dental students and dentists with local patients. On the other hand, language and knowledge of local risk factors were barriers for faculty members who were not local.

A limitation of the present study was that the participants were limited to the JDS faculty members, interns, and students, which may restrict the generalizability of the findings. However, some faculty members who had wide previous experiences with other health sectors participated in the FGDs as well. Also, limited participation of female faculty members was due to their limited numbers at JDS. However, quite equal participations of male and female interns and students were achieved. The interns and dental students provided insights regarding the effect of the clinical courses’ requirements on their oral cancer examination and patients’ education of oral cancer risk factors. Also, they provided insight into the aforementioned cultural barrier.

Further studies need to investigate the possible effect of the dental school curriculum and its clinical training requirements on the future oral cancer examination and patients’ education practice of dental students and dentists. Quantitative assessment of the relation between the cognitions of dentists and their behavior is required as well. For instance, investigation of the clinicians’ knowledge, attitude, subjective norms, and self-efficacy regarding oral cancer examination and patients’ education of oral cancer risk factors and observing the actual practice of oral cancer and patients’ education of its risk factors in the JDS clinics are essential steps to develop appropriate interventions.

## Conclusion

Broadly translated, our findings indicate that dental education and training at Jazan University Dental School are not covering/focusing on necessary related oral health issues and their risk factors of the community/region. Also, they cast a new light on the fact that dentists are not educating their patients because they lack the knowledge and the skill of health education and patients’ communication methods.

### Implications for Practice

Dental schools should empower their graduates through education and support to be able to contribute to reducing the local oral health burdens. In order for dental students to be competent in practicing oral cancer screening, the educational curriculum must emphasize on providing effective and necessary information and skills to perform oral cancer examination and patient education and to boost dental students’ capabilities through authentic and vicarious experiences by incorporating knowledge and skills training needed for oral cancer examination and patient education during all clinical academic years. This study would add to the technical ability of dental researcher to utilize different scientific approaches. For example, utilizing grounded theory to investigate the possible determinants and influences on dentists’ behavior and to develop a theory which could explain such behaviors. As well as, it would support in developing further studies and targeted interventions in Jazan Dental School and other dental schools in areas with similar circumstances of oral cancer high rates and utilization of smokeless tobacco, for example, in Sudan [25].

### Electronic Supplementary Material

Supplementary file 1 (Table 1). Coding process. A table that illustrates data coding process. (DOCX 18 kb)

Supplementary file 2 (Figure 1). A figure that illustrates the sequence of the methodology including participants’ distribution in the six focus groups discussions. (PDF 214 kb)

Supplementary file 3 (Figure 2). Coding tree. A figure that shows the generated coding tree from the data. (PDF 356 kb)

## Appendix 1. Clinicians’ interview guide

### Questions

1. Do you think that oral cancer is an issue in Jazan?
  - If yes, how /why? How serious is the problem?
  - If no: why?
2. What do you think about oral cancer examination/ screening?
  - In your opinion, do you think it is useful?
    - If yes, how /why?
    - If no: why?
3. What do you see as advantages of preforming an oral cancer examination/ screening?
4. What do you see as barriers of preforming an oral cancer examination/ screening?
  - Do you think you can do anything about it?
5. Do you think that there are clear guidelines/ regulations in Jazan dental school clinics for oral cancer examination/ screening?
  - If yes: do you think it is fallowed? If not: why?
  - If no: why?
6. What do you feel toward preforming an oral cancer examination/ screening for your patients in the dental clinic?
  - Do you think that you are comfortable of doing an oral cancer clinical examination/ screening?
    - If not: why?
7. In your opinion, what do you think about oral cancer training in your education?
  - How do you feel about the theoretical part of your education?
  - How do you feel about the practical/clinical part of your education?
  - What do you thing should be done to improve/strength the curriculum?
  - How could you contribute to improve/strength the overall experience of oral cancer clinical examination/ screening?
8. In your opinion, what do you think of dentists educating their patients about oral cancer cancers and their risk factors in the clinics?
  - In your opinion, do you think it is useful?
    - If yes, how /why?
    - If no: why?
9. In your opinion, certain risk factors of oral cancer such as alcohol and oral sex could be discussed with the patient.
  - If yes: is there any male/female differences and how you deal with it?
  - If no: why?
10. Do you have any thing that you would like to add?

## Appendix 2. Dental students’ and interns’ focus group guide

### Questions

1. What do you feel toward preforming an oral cancer examination/ screening for your patients in the dental clinic?
  - Do you think that you are comfortable of doing an oral cancer clinical examination/ screening?
    - If not: why?
2. In your opinion, what do you think about oral cancer training in your education?
  - How do you feel about the theoretical part of your education?
  - How do you feel about the practical/clinical part of your education?
  - What do you thing should be done to improve/strength the curriculum?
  - How could you contribute to improve/strength the overall experience of oral cancer clinical examination/ screening?
3. Do you think that there are clear guidelines/ regulations in Jazan dental school clinics for oral cancer examination/ screening?
  - If yes: do you think it is fallowed? If not: why?
  - If no: why? In your opinion, certain risk factors of oral cancer such as alcohol and oral sex could be discussed with the patient.
  - If yes: is there any male/female differences and how you deal with it?
  - If no: why?
4. What do you see as barriers of preforming an oral cancer examination/ screening?
  - Do you think you can do anything about it?
5. In your opinion, what do you think of dentists educating their patients about oral cancer cancers and their risk factors in the clinics?
  - In your opinion, do you think it is useful?
    - If yes, how /why?
6. Do you have any thing that you would like to add?

## References

[1] Petersen PE (2003). The World Oral Health Report 2003: continuous improvement of oral health in the 21st century – the approach of the WHO Global Oral Health Programme. Community Dent Oral Epidemiol.

[2] Saudi Cancer Registry. Saudi Arabia cancer incidence report 2010 [document on the Internet]. Riyadh, Saudi Arabia: Saudi Cancer Registry; 2014 [cited 2018 Apr 1]. Available from: http://ghdx.healthdata.org/organizations/saudi-cancer-registry

[3] Rodu B, Jansson C (2004). Smokeless tobacco and oral cancer: a review of the risks and determinants. Crit Rev Oral Biol Med.

[4] Allard WF, DeVol EB, Te OB (1999). Smokeless tobacco (Shamma) and oral cancer in Saudi Arabia. Community Dent Oral Epidemiol.

[5] Zhang X, Schmitz W, Gelderblom HR, Reichart PA (2001). Shammah-induced oral leukoplakia-like lesions. Oral Oncol.

[6] Castellsague X, Quintana MJ, Martinez MC, Nieto A, Sanchez MJ, Juan A (2004). The role of type of tobacco and type of alcoholic beverage in oral carcinogenesis. Int J Cancer.

[7] Al Agili DE, Park HK (2012). The prevalence and determinants of tobacco use among adolescents in Saudi Arabia. J Sch Health.

[8] Seoane J, Alvarez-Novoa P, Gomez I, Takkouche B, Diz P, Warnakulasiruya S et al (2016) Early oral cancer diagnosis: the Aarhus statement perspective. A systematic review and meta-analysis. Head Neck. 38(Suppl 1):E2182–E2189. 10.1002/hed.24050 10.1002/hed.24050 25783770

[9] Rodrigues PC, Miguel MC, Bagordakis E, Fonseca FP, de Aquino SN, Santos-Silva AR (2014). Clinicopathological prognostic factors of oral tongue squamous cell carcinoma: a retrospective study of 202 cases. Int J Oral Maxillofac Surg.

[10] Burzynski NJ, Rankin KV, Silverman S, Scheetz JP, Jones DL (2002). Graduating dental students’ perceptions of oral cancer education: results of an exit survey of seven dental schools. J Cancer Educ.

[11] Warnakulasuriya KA, Johnson NW (1999). Dentists and oral cancer prevention in the UK: opinions, attitudes and practices to screening for mucosal lesions and to counselling patients on tobacco and alcohol use: baseline data from 1991. Oral Dis.

[12] Andersen BL, Cacioppo JT (1995). Delay in seeking a cancer diagnosis: delay stages and psychophysiological comparison processes. Br J Soc Psychol.

[13] Grant E, Silver K, Bauld L, Day R, Warnakulasuriya S (2010). The experiences of young oral cancer patients in Scotland: symptom recognition and delays in seeking professional help. Bdj.

[14] Subramanian S, Sankaranarayanan R, Bapat B, Somanathan T, Thomas G, Mathew B, Vinoda J, Ramadas K (2009). Cost-effectiveness of oral cancer screening: results from a cluster randomized controlled trial in India. Bull World Health Organ.

[15] Ling W, Mijiti A, Moming A (2013). Survival pattern and prognostic factors of patients with squamous cell carcinoma of the tongue: a retrospective analysis of 210 cases. J Oral Maxillofac Surg.

[16] Tong A, Sainsbury P, Craig J (2007). Consolidated criteria for reporting qualitative research (COREQ): a 32-item checklist for interviews and focus groups. Int J Qual Health Care.

[17] Clovis JB, Horowitz AM, Poel DH (2002). Oral and pharyngeal cancer: practices and opinions of dentists in British Columbia and Nova Scotia. J Can Dent Assoc.

[18] Kujan O, Alzoghaibi I, Azzeghaiby S, Altamimi MA, Tarakji B, Hanouneh S, Idress M, Alenzi FQ, Iqbal M, Taifour S (2014). Knowledge and attitudes of Saudi dental undergraduates on oral cancer. J Cancer Educ.

[19] Decuseara G, MacCarthy D, Menezes G (2011). Oral cancer: knowledge, practices and opinions of dentists in Ireland. J Ir Dent Assoc.

[20] Haresaku S, Makino M, Sugiyama S, Naito T, Marino RJ (2018). Comparison of practices, knowledge, confidence, and attitude toward oral cancer among oral health professionals between Japan and Australia. J Cancer Educ.

[21] Fishbein M, Ajzen I (2011). Predicting and changing behavior: the reasoned action approach.

[22] Sanders AE, Lushington K (2002). Effect of perceived stress on student performance in dental school. J Dent Educ.

[23] Elani HW, Allison PJ, Kumar RA, Mancini L, Lambrou A, Bedos C (2014). A systematic review of stress in dental students. J Dent Educ.

[24] Almutairi KM (2015). Culture and language differences as a barrier to provision of quality care by the health workforce in Saudi Arabia. Saudi Med J.

[25] Warnakulasuriya S (2009). Global epidemiology of oral and oropharyngeal cancer. Oral Oncol.

