# Supplementary material for: What Do Dentists and Dental Students Think of Oral Cancer and Its Control and Prevention Strategies? A Qualitative Study in Jazan Dental School
Source: J Cancer Educ. 2019 Sep 10;36(1):134–42. doi: 10.1007/s13187-019-01609-z (PMC7835163; doi:10.1007/s13187-019-01609-z)
Supplement: Supplementary file 1 — (Table 1). Coding process. A table that illustrates data coding process. (DOCX 18 kb) [file 13187_2019_1609_MOESM1_ESM.docx]

| **Table 1 (Coding process)** |  |  |  |
| --- | --- | --- | --- |
| **Initial codes** | | **Focus codes** | **Theoretical codes** |
| - Fetal/Killing /Threatening disease - Predominating cancer - Specific geographical distribution - High prevalence cancer in the area specially among females - Affecting all ages | - Lacking specialized medical center - Discrete specialists - Delay diagnosis - Outreach program - Awareness of the issue | 1. Public Health Issue | Dental Education and training at Jazan University Dental School is not covering / focusing on necessary related oral health issues and their risk factors of the community/region.  Dentists are not educating their patients because they lack knowledge and skill on health education and patients’ communications methods |
| - Using Shamma - Most affected area related to Shamma in compare to the world - Combining Shamma with other risk factors; smoking, Ghat - High consumption of Shamma per day - Using Shamma is not limited by age or gender | - Starting using Shamma at young age - Culturally accepted - Using Ghat - Chewing Ghat is combined with smoking and using Shamma - Using “Toombak” - Pesticides used for Ghat | 1. Behavior and cultural related risk factors |  |
| - Curriculum building/ accreditation requirements - Courses sequencing - Lectures covering/focusing on region issues and needs - Clinical training on oral cancer - Exposure to actual cases - Protocol / guidelines | - Research data on the issue - Access to literature - Collaboration with other medical providers in relation to oral cancer - Patients’ education of related risk factor | 1. Curriculum influence |  |
| - Didactic and clinical training - Clinical exposure - Charts deficiency (not included in the chart) - Time consuming - Requirements focusing - Self interest - Enough knowledge - Specialty related issue | - Depending on previous oral exam - Clinical protocol/manual - Incentives / Individual benefit - Awareness of the region’s issue and risk factors - Responsibility - Supervision - Retaining patients | 1. Clinicians’ behavior toward oral cancer |  |
| - Cultural norms - Cultural differences - Socioeconomic - Gender - Stereotype - Language | - Knowledge - Age - Referring - Specialized center - Training | 1. Challenges and barriers |  |
