# Supplementary figures and images for: What Do Dentists and Dental Students Think of Oral Cancer and Its Control and Prevention Strategies? A Qualitative Study in Jazan Dental School
Source: J Cancer Educ. 2019 Sep 10;36(1):134–42. doi: 10.1007/s13187-019-01609-z (PMC7835163; doi:10.1007/s13187-019-01609-z)

**Figure 1 illustrating the methodology sequence**

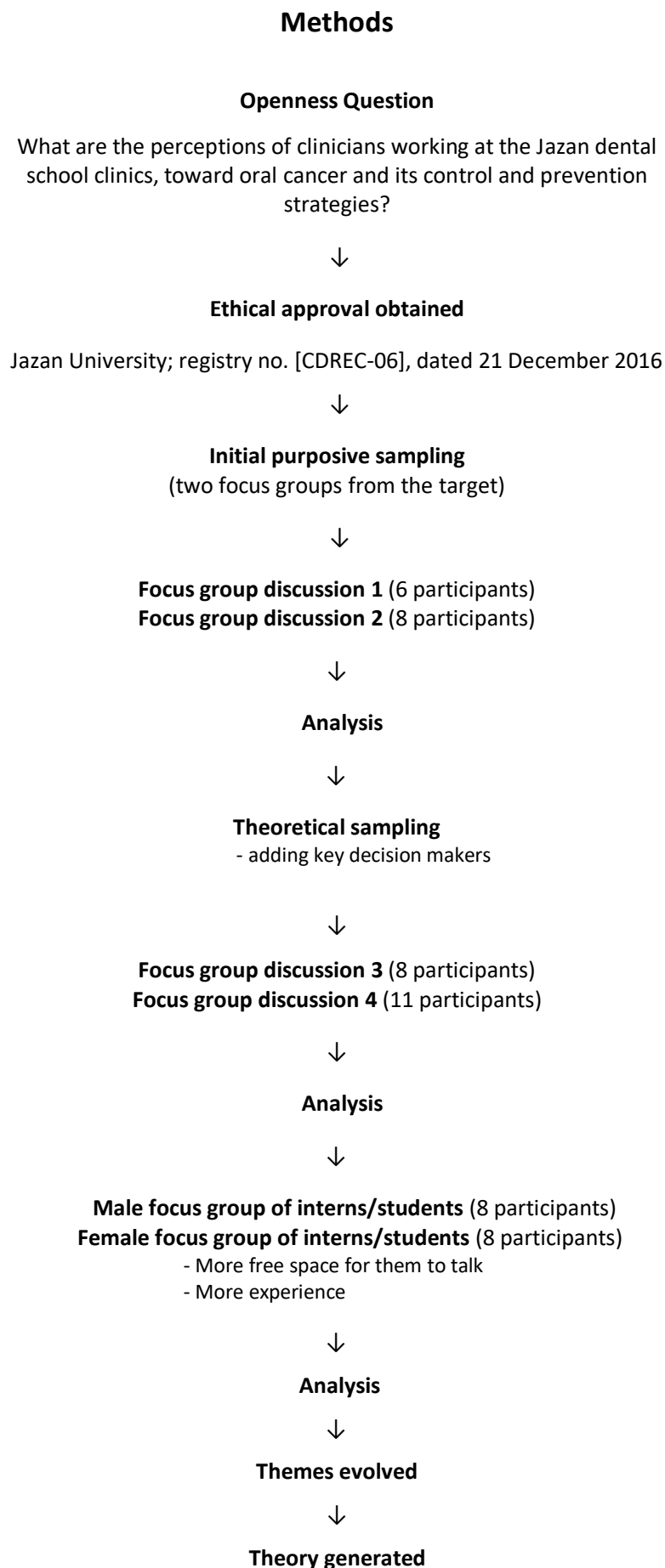

Supplement: Supplementary file 2 — (Figure 1). A figure that illustrates the sequence of the methodology including participants’ distribution in the six focus groups discussions. (PDF 214 kb) [file 13187_2019_1609_MOESM2_ESM.pdf]

Figure 2 (Coding Tree)

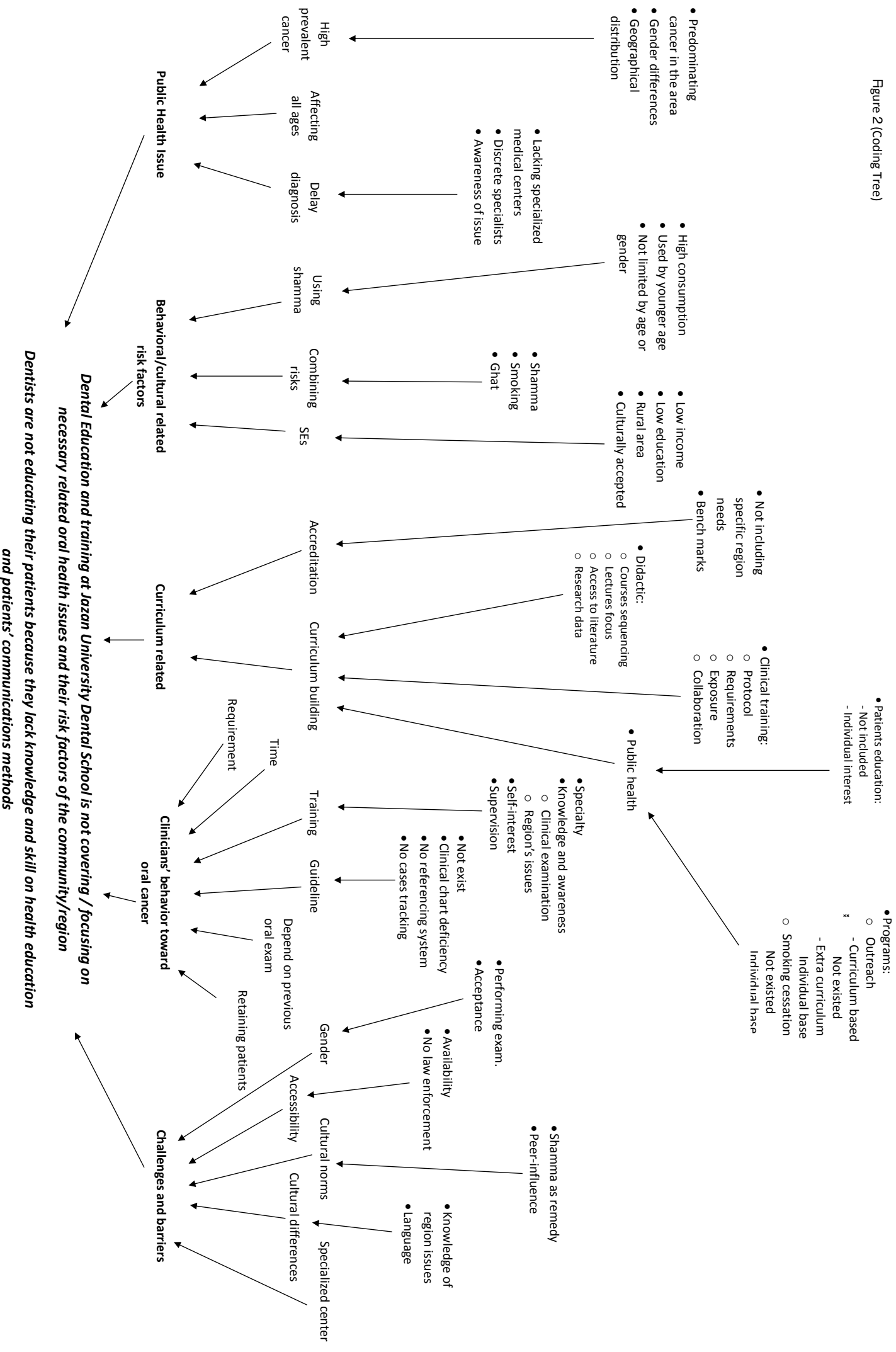

Supplement: Supplementary file 3 — (Figure 2). Coding tree. A figure that shows the generated coding tree from the data. (PDF 356 kb) [file 13187_2019_1609_MOESM3_ESM.pdf]
